# Supplementary material for: Photoactivated conductive MOF thin film arrays on micro-LEDs for chemiresistive gas sensing
Source: Nat Commun. 2025 Oct 30;16:9612. doi: 10.1038/s41467-025-64602-9 (PMC12575684; doi:10.1038/s41467-025-64602-9)
Supplement: Supplementary file 1 — Supplementary Information [file 41467_2025_64602_MOESM1_ESM.pdf]

Supplementary Information

Photoactivated Conductive MOF Thin Film Arrays on Micro-LEDs for Chemiresistive Gas Sensing

Kichul Lee [1,†], Young-Moo Jo[2,3,†*], Myung Sung Sohn[2,†], Mingyu Jeon[4], Cheolmin Kim[1], Osman Gul[1], Seon Ju Park[2], Ki Beom Kim[2], Ki Soo Chang[5,6], Chan Bae Jeong[5], Jihan Kim[4], Yun Chan Kang[2*], Inkyu Park[1*]

[1]Department of Mechanical Engineering, Korea Advanced Institute of Science and Technology (KAIST), Daejeon 34141, Republic of Korea

[2]Department of Materials Science and Engineering, Korea University, Seoul 02841, Republic of Korea

[3]School of Materials Science and Engineering, Kyungpook National University, Daegu 41566, Republic of Korea

[4]Department of Chemical and Biomolecular Engineering, Korea Advanced Institute of Science and Technology (KAIST), Daejeon 34141, Republic of Korea

[5]Center for Scientific Instrumentation, Korea Basic Science Institute, Daejeon 34133, Republic of Korea

[6]School of Electrical and Electronic Engineering, Chung-Ang University, Seoul 06974, Republic of Korea

† Equally contributed

[*]*Corresponding authors: Y.-M. Jo, Y. C. Kang, I. Park*

Email: jym754@knu.ac.kr, yckang@korea.ac.kr, inkyu@kaist.ac.kr

Contents

• Supplementary Notes 4–7

- Supplementary Note 1 | Supplementary experimental section (Materials and Characterization of materials) 4

- Supplementary Note 2 | Temperature measurement of the cMOF integrated on μLP. 5

- Supplementary Note 3 | The structure and training process of the e-nose system. 6,7

• Supplementary Figures 8–34

- Supplementary Fig. 1 | Scheme of measuring multiple gas sensors 8

- Supplementary Fig. 2 | PXRD patterns of Cu_3HHTP_2 powders 9

- Supplementary Fig. 3 | XPS spectra of CuHHTP-5C 10

- Supplementary Fig. 4 | SEM images of CuHHTP-*x*C at different coating cycles 11

- Supplementary Fig. 5 | Gas sensing transients of CuHHTP-*x*C sensors 12

- Supplementary Fig. 6 | Top-view SEM image and EDS analysis of CuHHTP-1C, CuHHTP-3C, and CuHHTP-5C 13

- Supplementary Fig. 7 | Normalized sensing transients of CuHHTP-*x*C sensors 14

- Supplementary Fig. 8 | SEM images of NiHHTP-*y*C/CuHHTP-5C and CoHHTP-*y*C/CuHHTP-5C layers 15

- Supplementary Fig. 9 | Energy-dispersive X-ray spectroscopy (EDS) mapping of NiHHTP-7C/CuHHTP-15C 16

- Supplementary Fig. 10 | Energy-dispersive X-ray spectroscopy (EDS) mapping of CoHHTP-7C/CuHHTP-15C 17

- Supplementary Fig. 11 | XPS spectra of NiHHTP-7C/CuHHTP-5C 18

- Supplementary Fig. 12 | XPS spectra of CoHHTP-7C/CuHHTP-5C 19

- Supplementary Fig. 13 | Gas sensing transients of NiHHTP-*y*C/CuHHTP-5C sensors 20

- Supplementary Fig. 14 | Gas sensing transients of CoHHTP-*y*C/CuHHTP-5C sensors 21

- Supplementary Fig. 15 | Normalized sensing transients of NiHHTP-*y*C/CuHHTP-5C sensors to NO_2 22

- Supplementary Fig. 16 | Fabrication process of the photoactivated μLED gas sensor integrated with cMOF 23

- Supplementary Fig. 17 | Repeatability of the CuHHTP-15C sensor to EtOH under external UV light source 24

- Supplementary Fig. 18 | Raman spectroscopy results of CuHHTP-15C film of before and after EtOH repeatability test under UV light 25

- Supplementary Fig. 19 | Evaluation of the μLED's L-I-V characteristics 26

- **Supplementary Fig. 20 |** Gas sensing results for each target gas using optimized cMOF layers under varied μLED conditions 27

- **Supplementary Fig. 21 |** Comparative study of the recovery behavior of the CuHHTP-5C sensor toward NO_2 under thermal and photoactivation conditions 28

- **Supplementary Fig. 22 |** Repeatability test of optimized cMOF sensors under cyclic gas exposures 29

- **Supplementary Fig. 23 |** UV-vis measurement and Tauc plot of CuHHTP-*x*C and CoHHTP-7C sensors 30

- **Supplementary Fig. 24 |** Temperature measurement of cMOF integrated on μLP using IR camera 31

- **Supplementary Fig. 25 |** Calibrated temperature profiles of blue μLED and Cu_3HHTP_2-synthesized blue μLED samples under continuous L2 illumination. 32

- **Supplementary Fig. 26 |** Data augmentation process for gas response data of cMOF sensor array 33

- **Supplementary Fig. 27 |** Structure of the e-nose system based on a chemiresistive cMOF sensor array. 34

• **Supplementary Table** 35–36

- **Supplementary Table 1 |** Summary of CuHHTP-*x*C response and sample-to-sample deviations across coating cycles 35

- **Supplementary Table 2 |** Prediction errors (mean absolute error; MAE) of the CNN model for gas types and concentrations. 36

Supplementary Note 1 | Supplementary experimental section (Materials and Characterization of materials).

Materials. 2,3,4,7,10,11-Hexahydroxytriphenylene (H_6HHTP; >95.0%; TCI chemical), copper(II) acetate hydrate (99.99%; Sigma-Aldrich), cobalt(II) acetate tetrahydrate (98.0%; Junsei Chemical), and nickel(II) acetate tetrahydrate (98.0%; Junsei Chemical) were employed for the synthesis of cMOFs. All reagents were used without further purification.

Characterization of materials. Scanning Electron Microscopy (SEM) analysis was conducted using the JEOL JSM-IT500HR Field Emission Scanning electron microscope, utilizing SED detection, and operated at a voltage of 10.00 kV. Raman spectroscopy was measured using a Renishaw Raman microscope, utilizing a 532 nm laser beam at 1 mW beam. The absorbance of cMOFs was analyzed using a UV−Vis spectrophotometer (Lambda 1050, PerkinElmer, USA). The aforementioned materials were deposited on 1×1 cm^2 transparent quartz substrates for the absorbance measurement. X-ray photoelectron spectroscopy (XPS) was measured using Thermofisher Scientific Nexsa G2 X-ray photoelectron spectrometer, utilizing 2 kV ion energy Al Kα X-ray source with 400 μm beam size.

Supplementary Note 2 | Temperature measurement of the cMOF integrated on μLP.

An infrared micro-thermography system (SC5000 camera, FLIR, USA) was used to verify whether the surface temperature of the cMOF on the μLP increases due to the photothermal effect. Two samples were prepared: a blue μLP and a blue μLP with CuHHTP-5C deposited on top. Supplementary Fig. 24 illustrates the experimental setup. In the experimental procedure, the samples were mounted on a thermoelectric cooler (TEC) plate, which allowed for precise temperature control. This was connected to an electrical probing system. The spatial infrared sensor signal, denoted as $I_s[T(x,y)]$, can be expressed as Eq. (1)

$$I_s[T(x,y)]=R(x,y)\{\varepsilon_s(x,y)\cdot I_{bb}[T(x,y)]+I_{reflect}(x,y)+I_{surr}(x,y)\} \quad (1)$$

Where $R(x,y)$ is the spatial response variation of the instrument, ε_s is the emissivity distribution of the sample, and I_{bb} refers to Planck's blackbody radiation. $I_{reflect}$ represents reflected radiation from the sample, while I_{surr} corresponds to the ambient background radiations. Prior to measurement, a calibration was performed to correct errors caused by uncertainty in the emissivity (ε_s) and reflectivity ($I_{reflect}$) of the surface materials of the μLP, such as contact metals (gold), indium tin oxide, GaN, and SiO_2, as well as unwanted ambient radiation (I_{surr}). Calibration was performed using the dummy μLP chips and black paint with a high emissivity of 0.96 as references, while a resistance temperature detector (RTD) sensor was employed to precisely measure the temperature of the dummy μLP chips. As a result, the actual temperature of the emitting μLED was accurately calculated. The measurements were taken while increasing the forward bias of the μLP samples from 1 V (OFF-state) to 4 V. Both the cMOF-integrated μLP and the non-integrated μLP exhibited a surface temperature increase of less than 0.5°C. The blue μLP mainly used in this study corresponds to the L2 condition, which operates at a forward bias of 3.1 V. Under this condition, the μLP exhibited negligible temperature increase even after continuous operation for 1 hour (Supplementary Fig. 25). Thus, this analysis confirms that gas sensing in the cMOF integrated on the μLED is solely driven by photoactivation, with no contribution from the μLED's photothermal effect.

Supplementary Note 3 | The structure and training process of the e-nose system

In this study, the e-nose system is composed of a 4-cMOF sensor array and a convolutional neural network (CNN)-based deep learning algorithm. Each individual cMOF sensor generates a temporal resistance signal, where the *x*-axis represents time and the *y*-axis represents the signal response, forming a 1 × (time) matrix. Since all sensors were operated simultaneously, the signals were concatenated along the vertical axis to construct a 4 × (time) matrix. The collected signals were then normalized to R_g/R_a and structured into a 4 (number of sensors) × 7000 seconds (gas test duration) matrix. The detailed CNN architecture is shown in Supplementary Fig. 27.

CNNs are particularly well-suited for tasks that require the extraction of spatially or temporally correlated features from structured data. Unlike fully connected deep neural networks (DNNs) that flatten input matrices and lose local correlation information, CNNs are able to preserve and exploit the spatial (or temporal) structure of input data, making them highly effective for analyzing time-dependent signals. In our system, the transient signals of the sensor over time exhibit uniquely different profiles for each gas and carry critical information. CNNs are advantageous in this context because they can recognize these differences, which are difficult to capture using only static features such as the response magnitude (e.g., R_g/R_a at the maximum point of the gas response).

To preprocess the data, a 60-second sliding time window moving at 1-second intervals was applied, converting the original 4 × 7000 matrix into 6940 matrices of size 4 × 60. This sliding window approach enables the model to not only analyze steady-state response magnitudes (R_g/R_a) but also to capture unique dynamic features such as the initial slope, rise shape, and transient fluctuations specific to each gas. As a result, the system's ability to distinguish between gases is significantly enhanced beyond simple magnitude-based classification. To enhance the diversity of the training data, we first applied data augmentation to the original dataset (Supplementary Fig. 26). The augmented data were used exclusively for training the CNN model. The original (non-augmented) data were then randomly split in a 1:1 ratio to construct the validation and test sets. No overlap exists between the training, validation, and test sets, thereby ensuring a strict separation and avoiding data leakage.

A 2D convolutional layer with a 4 × 30 filter size and 6 output channels was applied, using a stride of 2 seconds. Each convolutional layer was followed by batch normalization (BN) and a leaky rectified linear unit (leaky-ReLU) activation function to improve training stability and generalization. After convolution, the extracted features were passed through three fully connected (FC) layers consisting of 32, 16, and 8 nodes, respectively, each also employing batch normalization and leaky-ReLU activation.

The final output structure was designed for dual tasks: gas classification and concentration prediction. The classification output consisted of 5 nodes corresponding to air, EtOH, TMA, NH_3, and NO_2 classes, using a softmax function to predict the gas label with the highest probability. The regression head included 1 node to predict gas concentration. For improved robustness and accuracy of concentration predictions, the TRIMMEAN function was applied as post-processing within the 60-second time window, excluding the top and bottom 20% of values to minimize the effect of outlier fluctuations.

The CNN model was trained by minimizing a combined loss function, defined as Eq. (2).

$$L_{\text{total}} = w \cdot L_{\text{cross-entropy}} + (1 - w) \cdot L_{\text{MSE}} \; (w = \text{weight}) \tag{2}$$

where $L_{\text{cross-entropy}}$ represents the categorical cross-entropy loss for classification, L_{MSE} is the mean squared error for regression, and w is the weighting factor between the two tasks. The Adam optimizer was used for training with a learning rate (Lr), $\eta = 10^{-4}$. Through hyperparameter tuning, the final values were set as $w = 0.05$ and epochs = 2900. Other tuned hyperparameters included the batch size, stride, number of filters, and kernel size to optimize model performance.

Importantly, the CNN approach led to two major improvements compared to using only the sensor responses and traditional machine learning techniques:

(1) It significantly enhanced gas selectivity by leveraging time-dependent dynamic features (transient response) rather than relying solely on response magnitude (R_g/R_a).

(2) It dramatically reduced the prediction latency, enabling gas type and concentration to be identified within about one minute. Including the initial sliding window accumulation, the total decision time stayed under two minutes, which is still substantially faster than the intrinsic

response and recovery times of the cMOF sensors (typically tens of minutes, as shown in Supplementary Fig. 20).

Overall, the CNN-based e-nose system presented in this study provides highly selective, fast, and accurate real-time gas prediction.

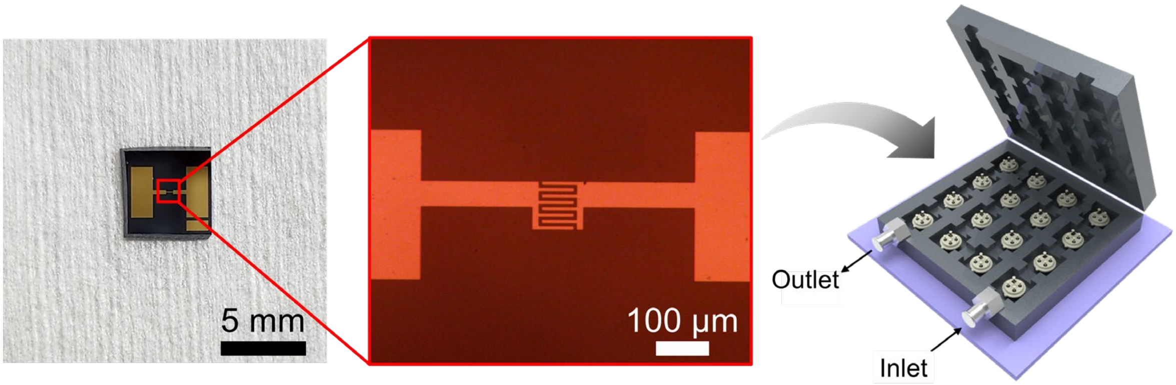

Supplementary Fig. 1 | Au-interdigitated electrodes (IDEs) on bare Si substrates and a home-made gas sensing chamber that simultaneously measures multiple gas sensors.

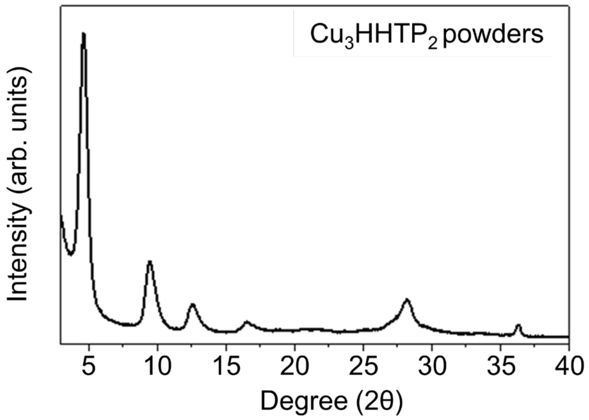

Supplementary Fig. 2 | Powder X-ray diffraction (PXRD) patterns of Cu_3HHTP_2 powders.

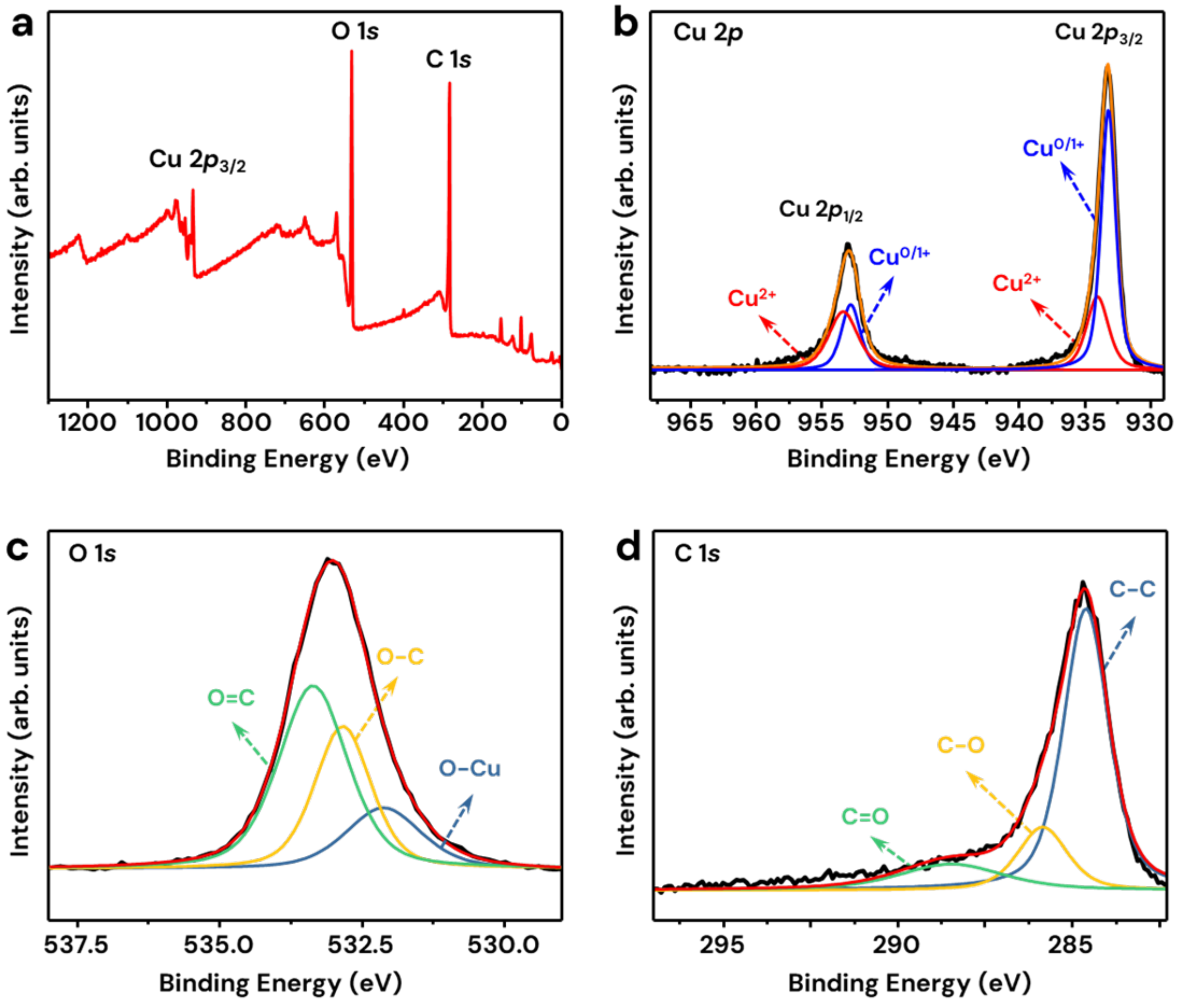

Supplementary Fig. 3 | a–d XPS spectra of CuHHTP-5C: (**a**) Survey scan, (**b**) Cu 2*p*, (**c**) O 1*s*, and (**d**) C 1*s*.

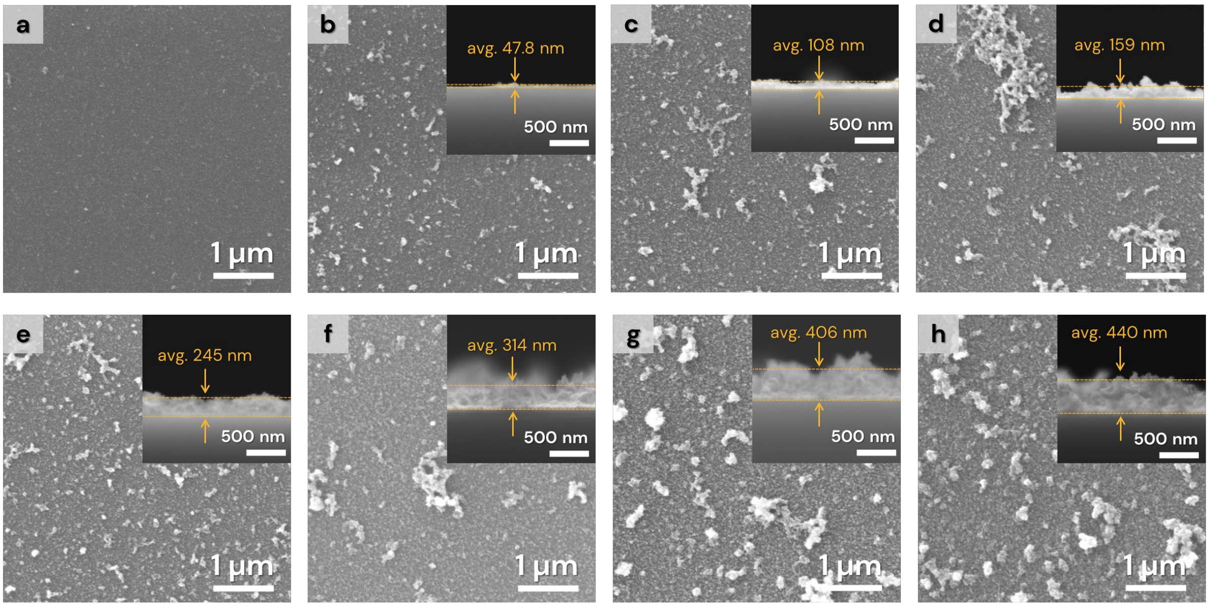

Supplementary Fig. 4 | a–h SEM images of CuHHTP-*x*C at different coating cycles (*x*= 1—15): (**a**) 1C, (**b**) 3C, (**c**) 5C, (**d**) 7C, (**e**) 9C, (**f**) 11C, (**g**) 13C, and (**h**) 15C. The CuHHTP-1C films were too thin to be measured accurately, and the CuHHTP-3C films exhibited discontinuities in the cross-sectional view. (The thickness of thin film was measured 10 times to calculate average.)

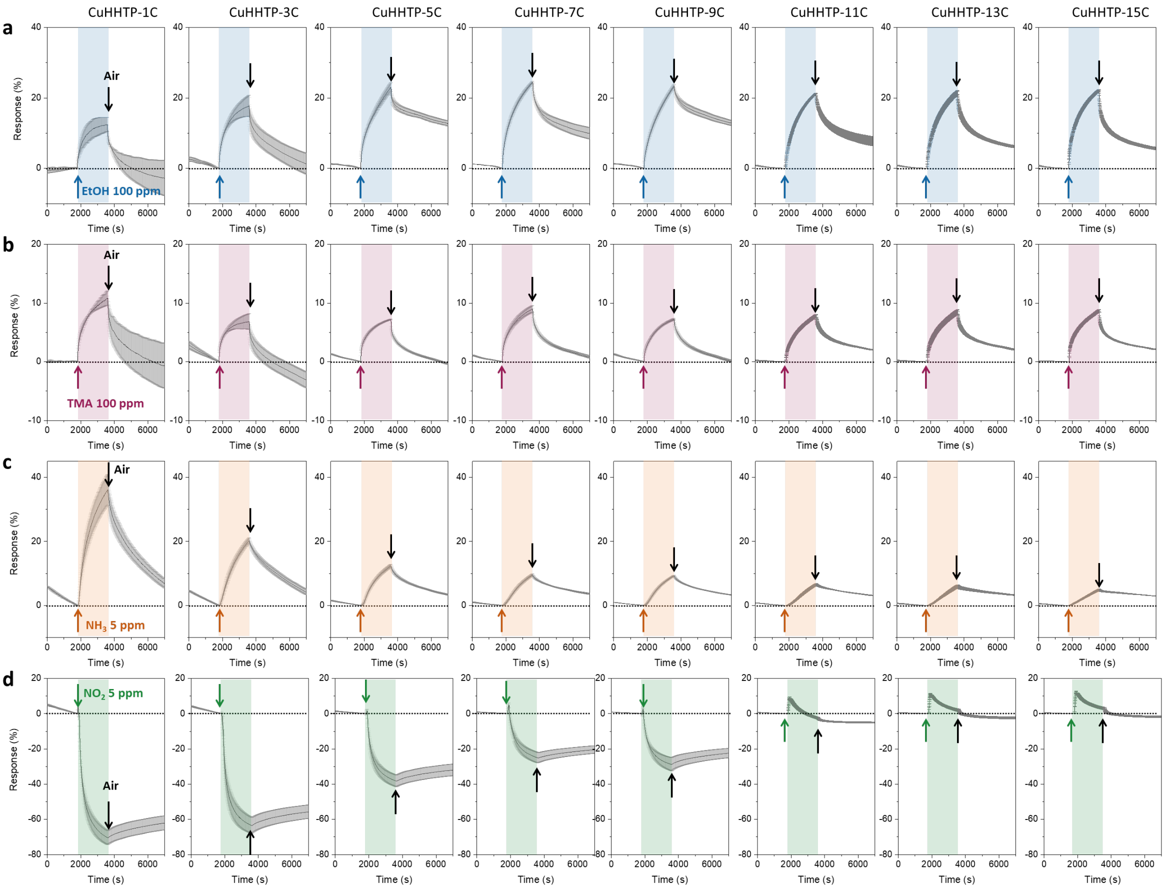

Supplementary Fig. 5 | a–d Gas sensing transients of CuHHTP-*x*C sensors (*x* = 1, 3, 5, 7, 9, 11, 13, and 15) to (**a**) 100 ppm ethanol (EtOH), (**b**) 100 ppm trimethylamine (TMA), (**c**) 5 ppm ammonia (NH_3), and (**d**) 5 ppm nitrogen dioxides (NO_2), under dark conditions. All results represent the average values from different sensors (n= 2 to 4, shading: standard deviation).

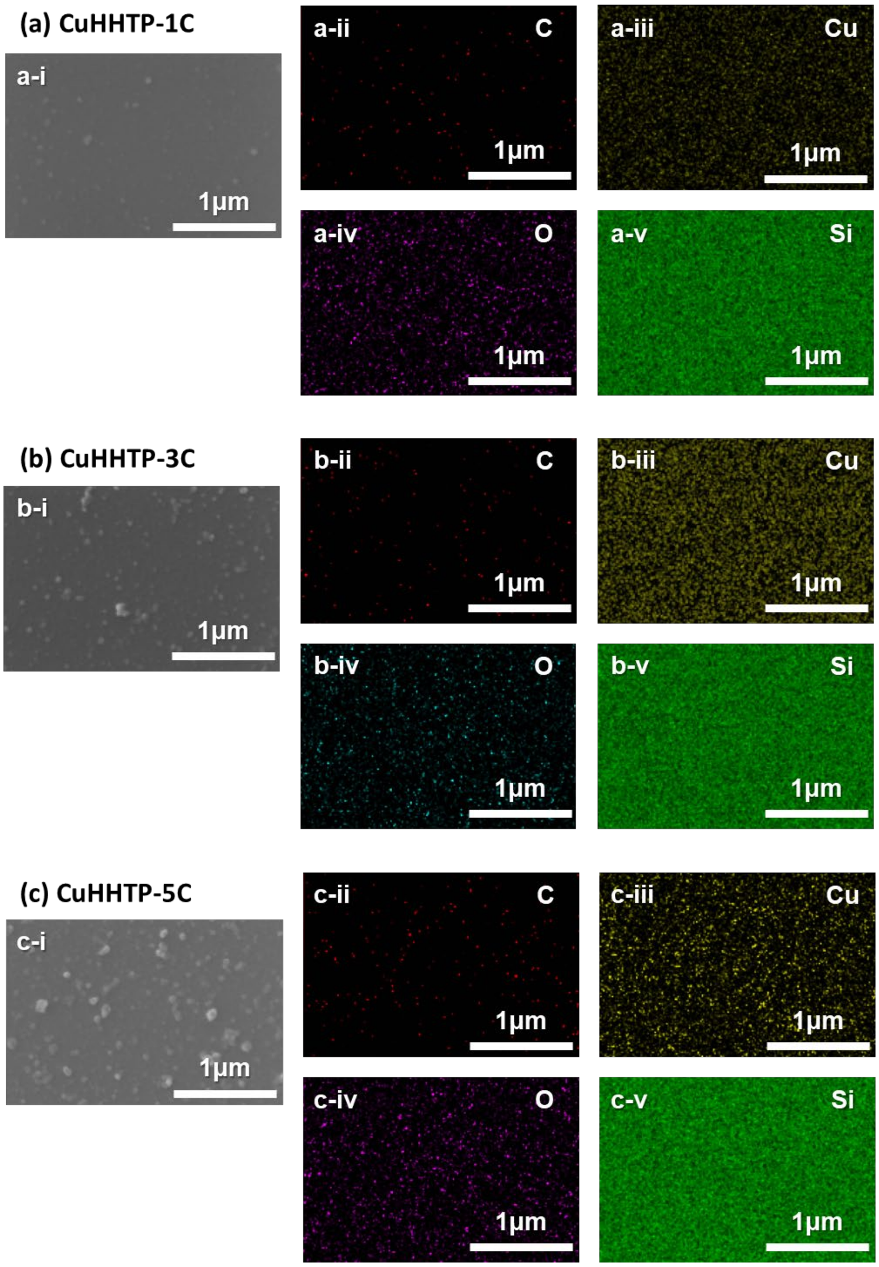

Supplementary Fig. 6 | a–c Top-view SEM image and EDS analysis of CuHHTP-1C, CuHHTP-3C, and CuHHTP-5C. (**i**) Top view SEM image of CuHHTP-5C on Si substrate. (**ii–v**) EDS mapping of CuHHTP-5C on Si substrate; (**ii**) C element, (**iii**) Cu element, (**iv**) O element, and (**v**) Si element. The particles on the film surfaces are confirmed as the agglomerated Cu_3HHTP_2 particles. These particles are grown through the rapid LBL process to optimize the engineering process, but this does not critically affect the gas sensing properties, as the batch-to-batch deviations remain below 10% (see Supplementary Table 1).

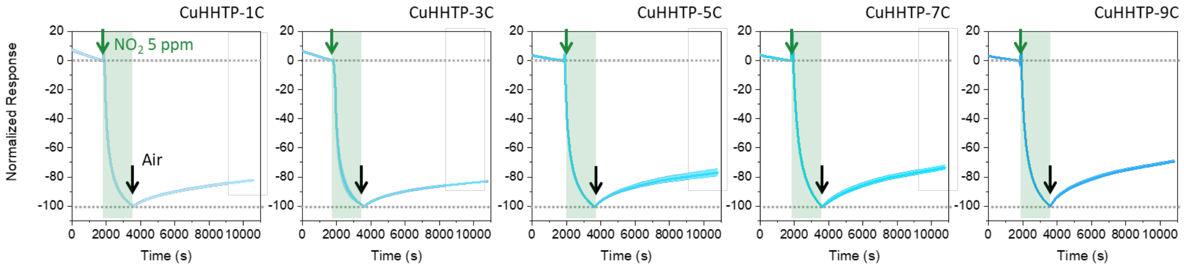

Supplementary Fig. 7 | Normalized sensing transients of CuHHTP-*x*C sensors (*x* = 1, 3, 5, 7, and 9) to 5 ppm NO_2. Thick samples could not be normalized due to low sensitivity and fluctuations in the transient response. All results represent the average values from different sensors (n= 2 to 4, shading: standard deviation).

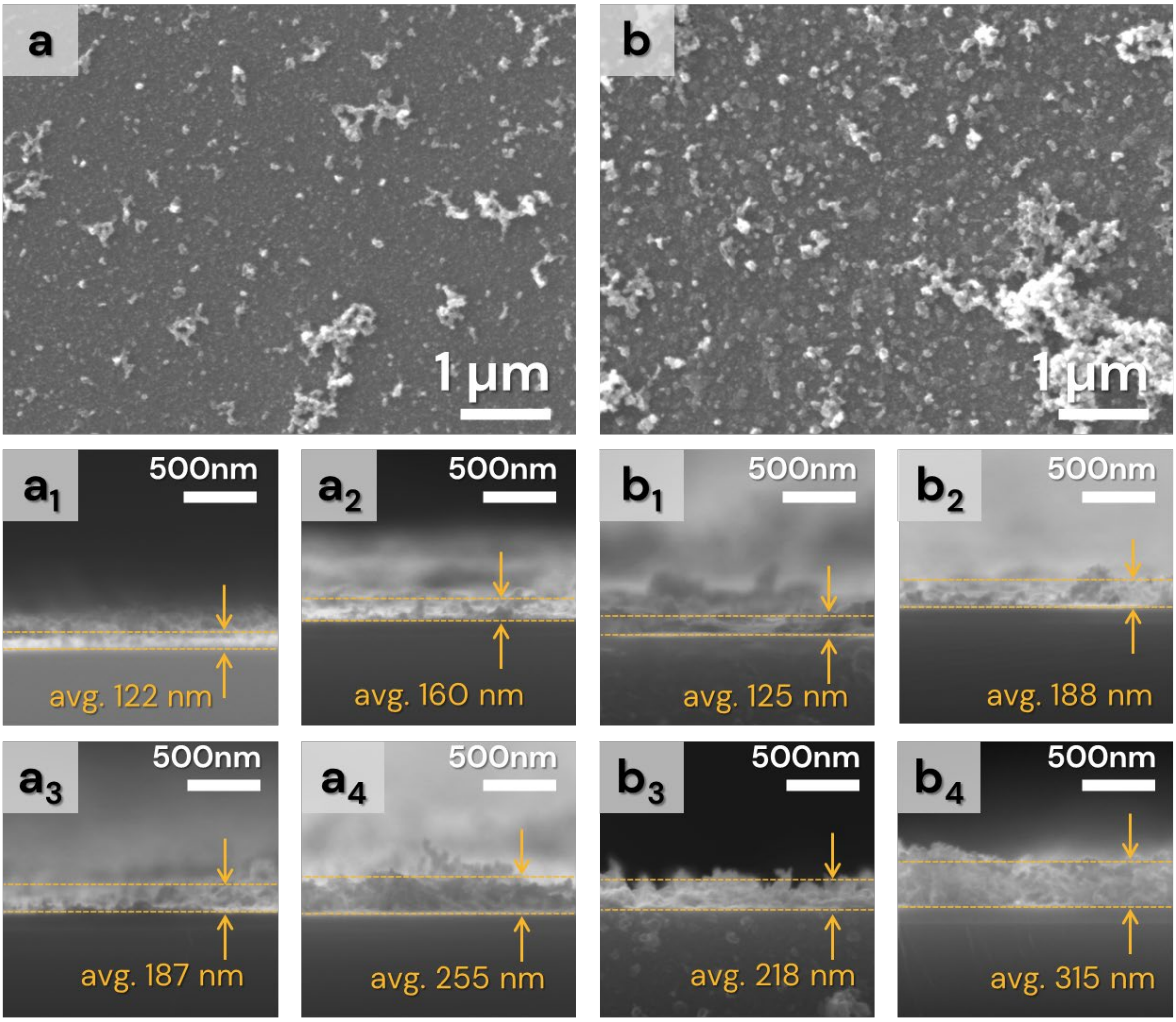

Supplementary Fig. 8 | a–b Top view SEM images of (**a**) NiHHTP-7C/CuHHTP-5C and (**b**) CoHHTP-7C/CuHHTP-5C. Cross-section view SEM images of (**a**; a_1—a_4) NiHHTP-*y*C/CuHHTP-5C (*y*=1,3,5,7) and (**b**; b_1—b_4) CoHHTP-*y*C/CuHHTP-5C (*y*=1,3,5,7). (The thickness of thin film was measured 10 times to calculate average.)

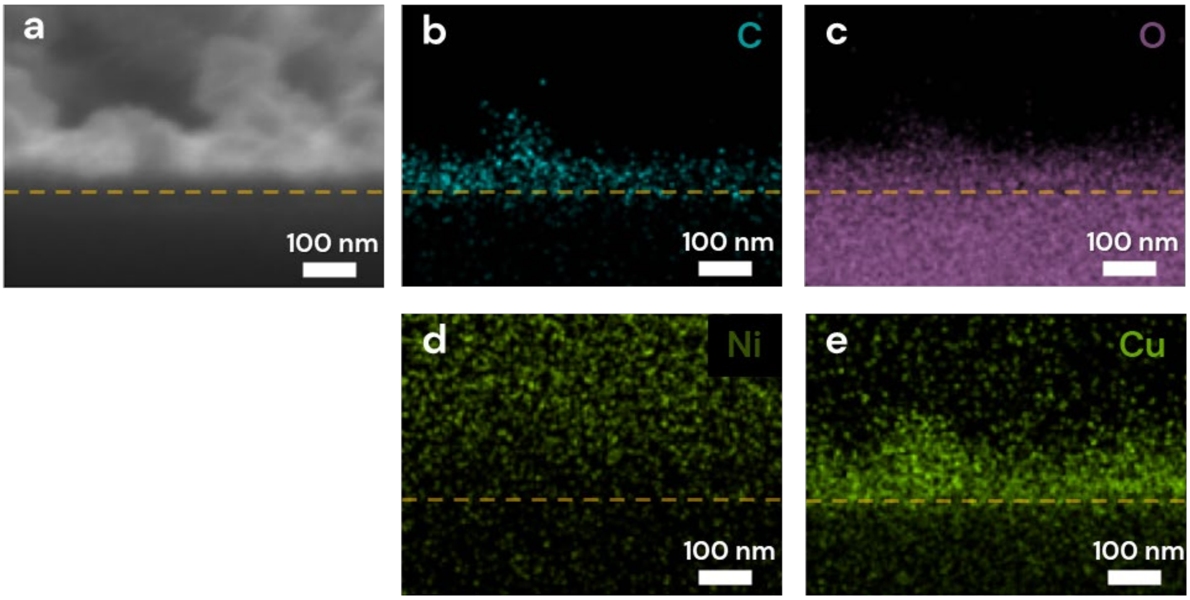

Supplementary Fig. 9 | a Cross-section view SEM image of NiHHTP-7C/CuHHTP-15C on Si/SiO_2 substrate. **b–e** Energy-dispersive X-ray spectroscopy (EDS) mapping of NiHHTP-7C/CuHHTP-15C on Si/SiO_2 substrate; (**b**) C element, (**c**) O element, (**d**) Ni element, and (**e**) Cu element. The substrate is tilted by 3° to observe the presence of both the Ni element in the overlayers and the Cu element in the base layer.

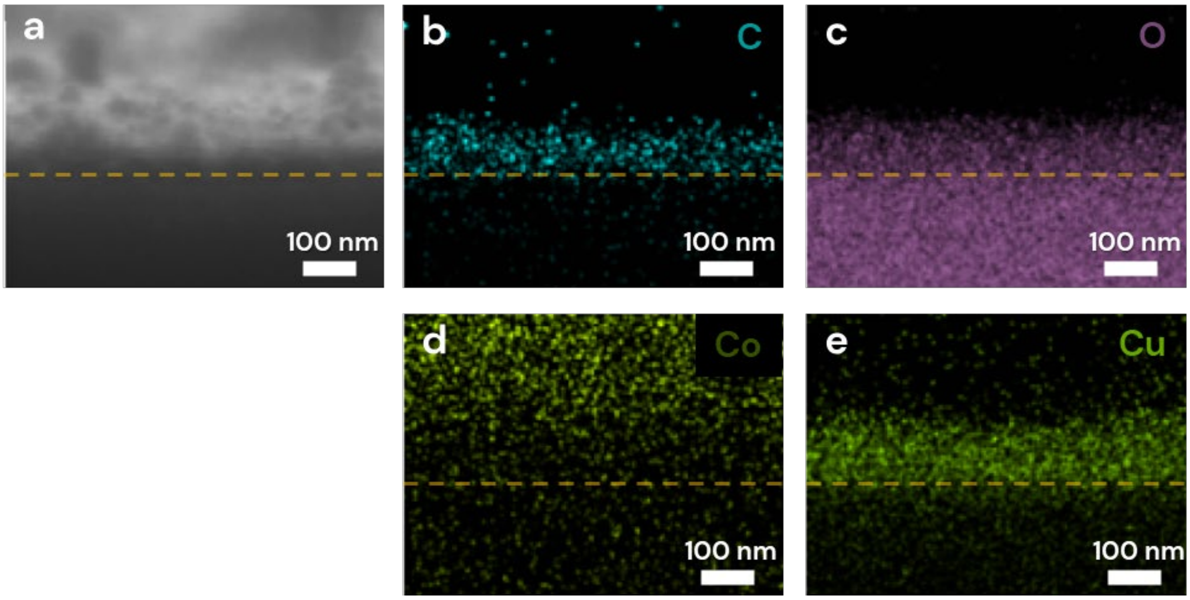

Supplementary Fig. 10 | a Cross-section view SEM image of CoHHTP-7C/CuHHTP-15C on Si/SiO_2 substrate. **b–e** Energy-dispersive X-ray spectroscopy (EDS) mapping of CoHHTP-7C/CuHHTP-15C on Si/SiO_2 substrate; (**b**) C element, (**c**) O element, (**d**) Co element, and (**e**) Cu element. The substrate is tilted by 3° to observe the presence of both the Co element in the overlayers and the Cu element in the base layer.

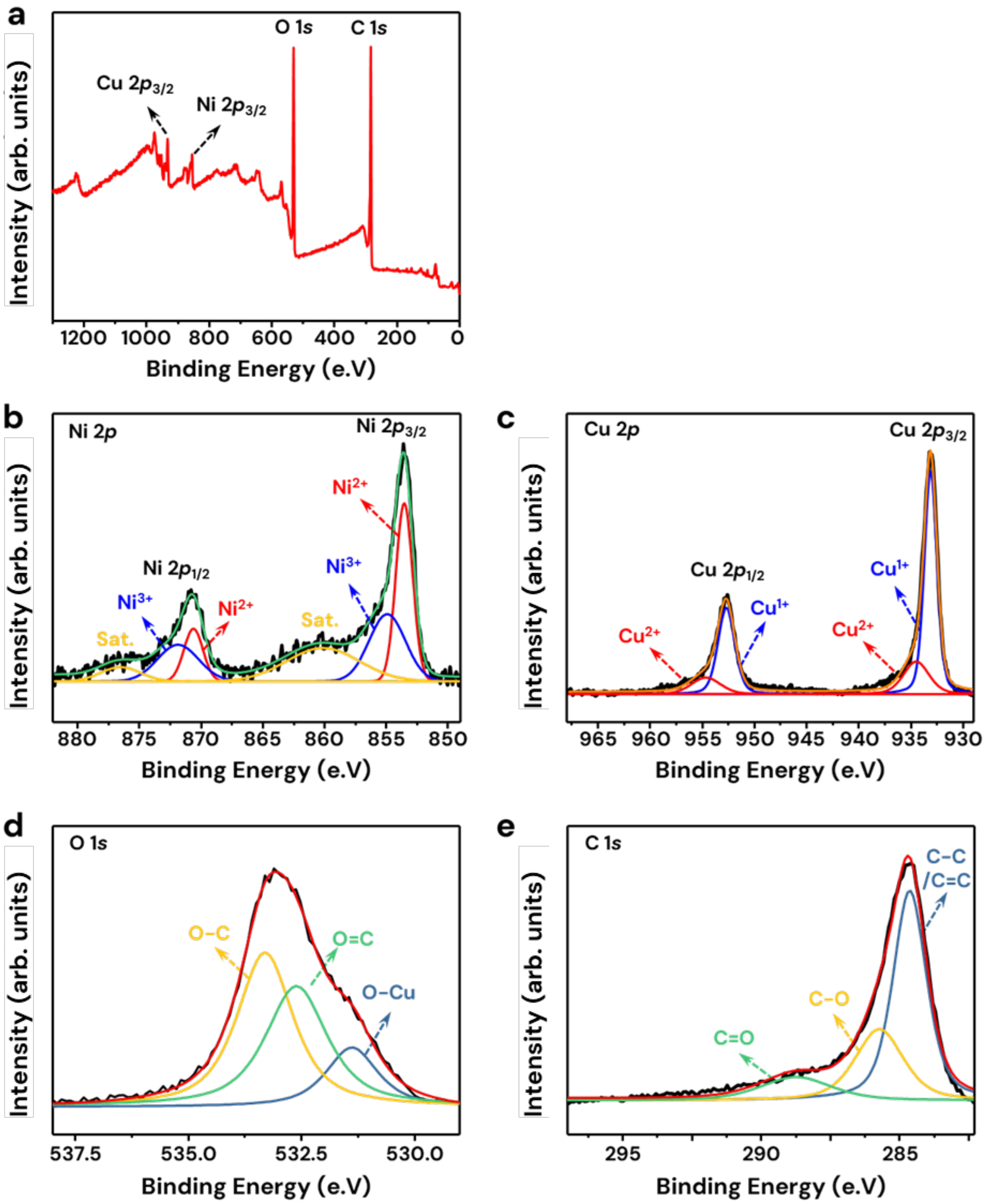

Supplementary Fig. 11 | a–e XPS spectra of NiHHTP-7C/CuHHTP-5C: (**a**) Survey scan, (**b**) Ni 2*p*, (**c**) Cu 2*p*, (**d**) O 1*s*, and (**e**) C 1*s*.

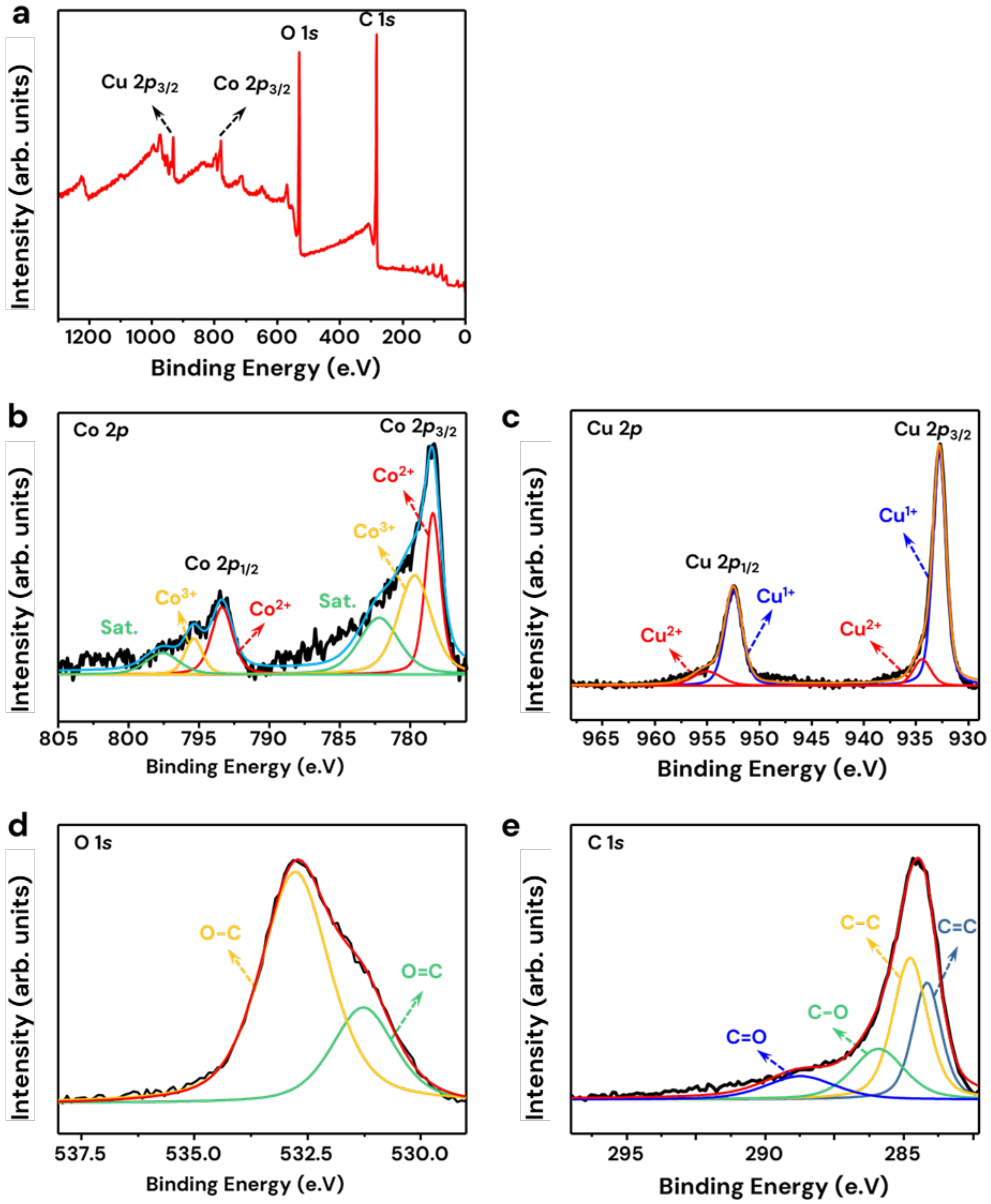

Supplementary Fig. 12 | a–e XPS spectra of CoHHTP-7C/CuHHTP-5C: (**a**) Survey scan, (**b**) Co 2*p*, (**c**) Cu 2*p*, (**d**) O 1*s*, and (**e**) C 1*s*.

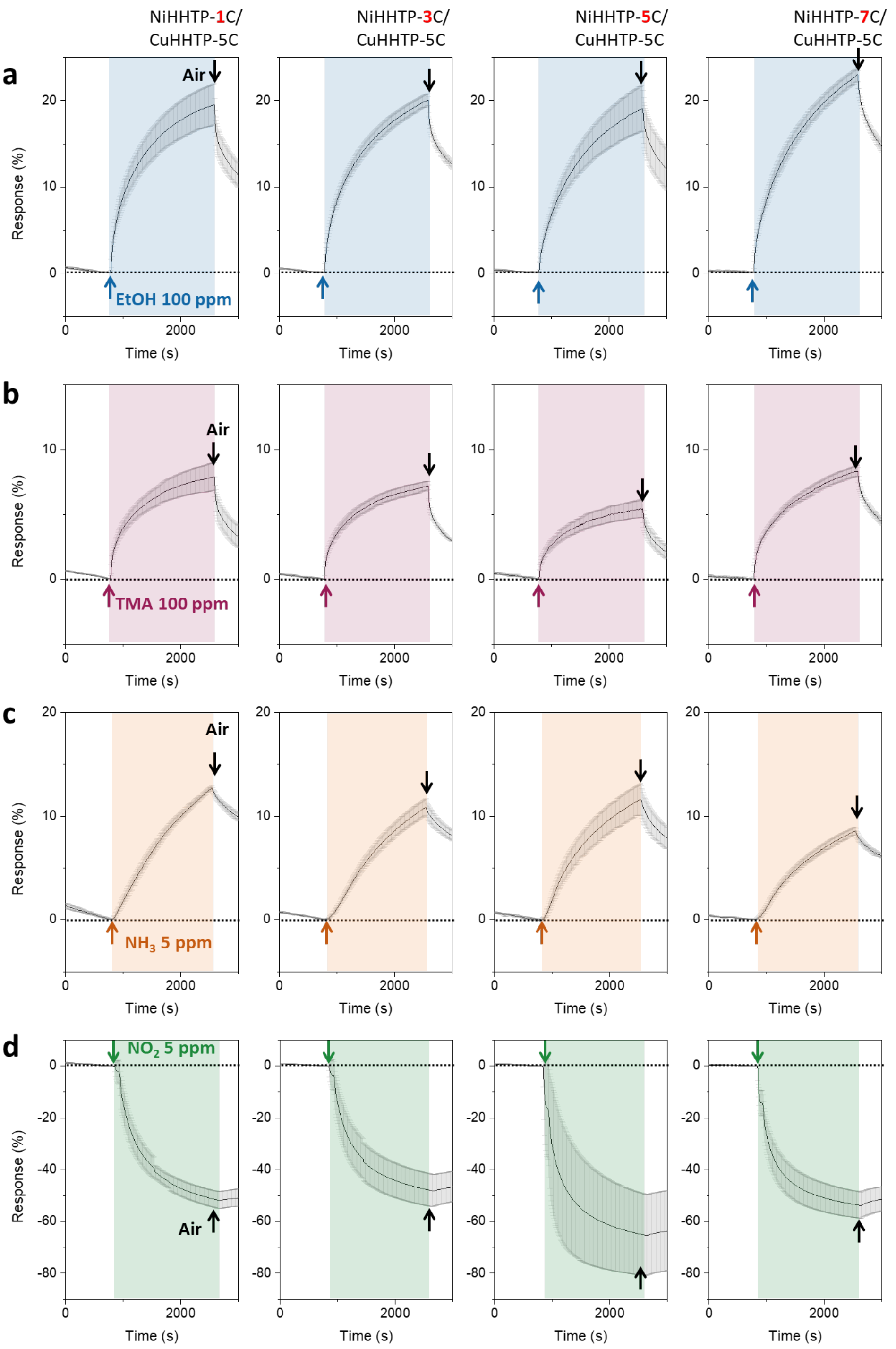

Supplementary Fig. 13 | a–d Gas sensing transients of NiHHTP-*y*C/CuHHTP-5C sensors (*y* = 1, 3, 5, and 7) to (**a**) 100 ppm ethanol (EtOH), (**b**) 100 ppm trimethylamine (TMA), (**c**) 5 ppm ammonia (NH_3), and (**d**) 5 ppm nitrogen dioxides (NO_2), under dark conditions. All results represent the average values from different sensors (n= 2 to 4, shading: standard deviation).

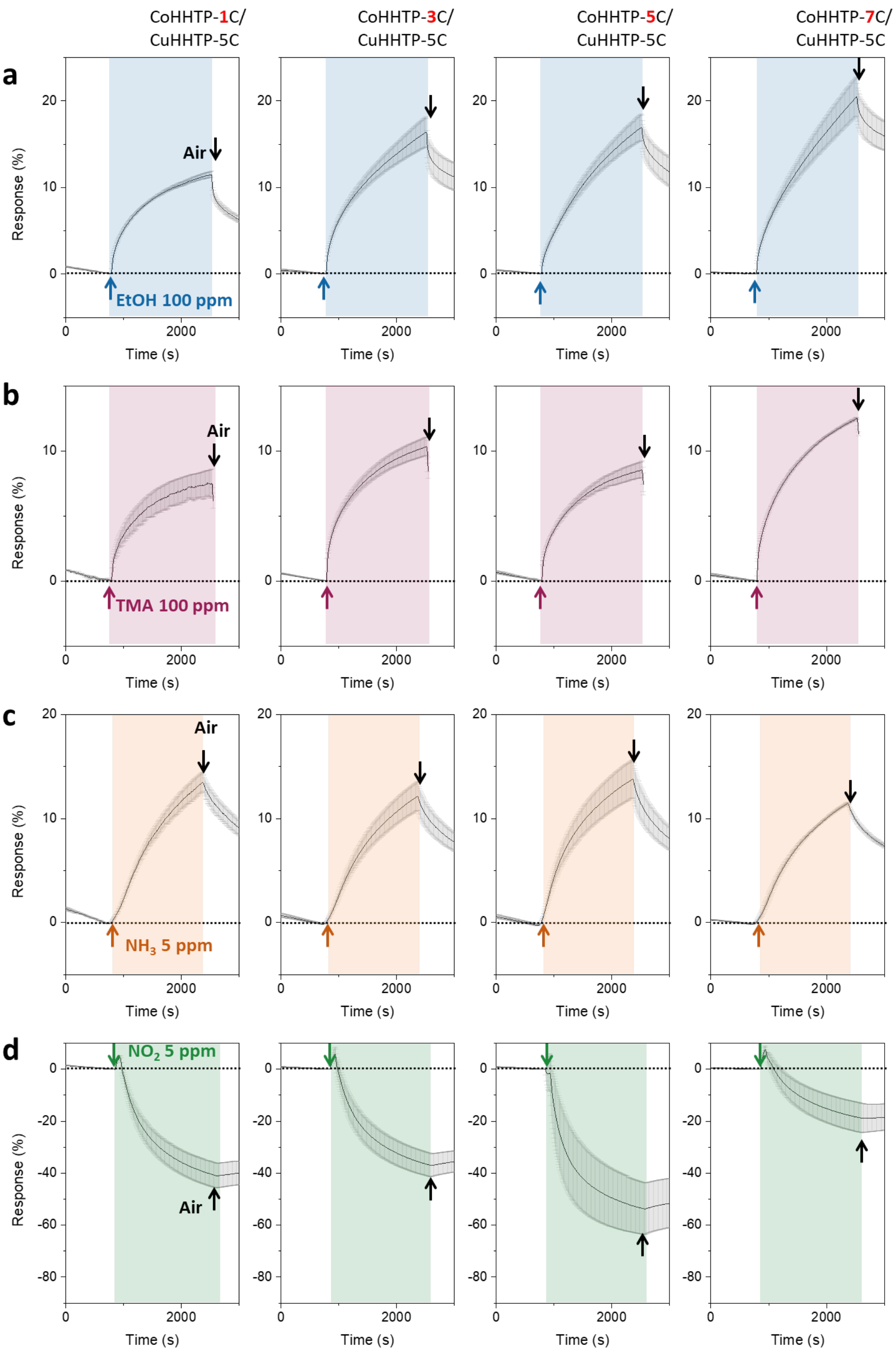

Supplementary Fig. 14 | a–d Gas sensing transients of CoHHTP-*y*C/CuHHTP-5C sensors (*y* = 1, 3, 5, and 7) to (**a**) 100 ppm ethanol (EtOH), (**b**) 100 ppm trimethylamine (TMA), (**c**) 5 ppm ammonia (NH_3), and (**d**) 5 ppm nitrogen dioxides (NO_2), under dark conditions. All results represent the average values from different sensors (n= 2 to 4, shading: standard deviation).

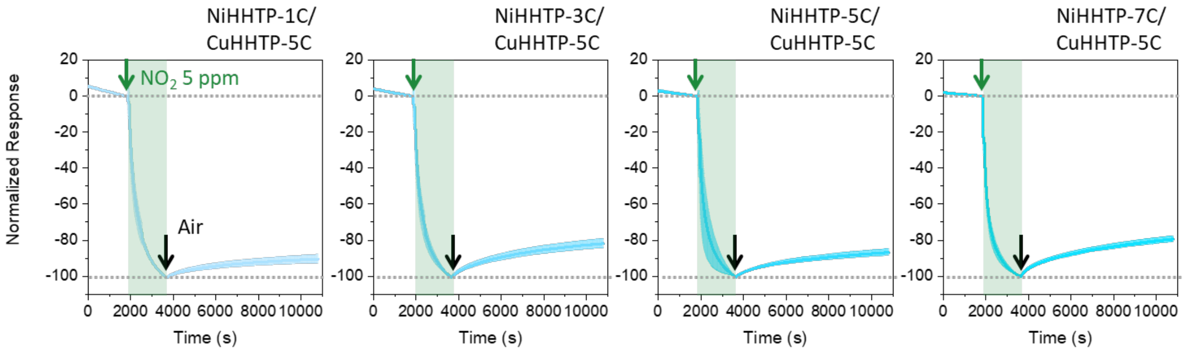

Supplementary Fig. 15 | Normalized sensing transients of NiHHTP-*y*C/CuHHTP-5C sensors (*y* = 1, 3, 5, and 7) to 5 ppm NO_2. All results represent the average values from different sensors (n= 2 to 4, shading: standard deviation).

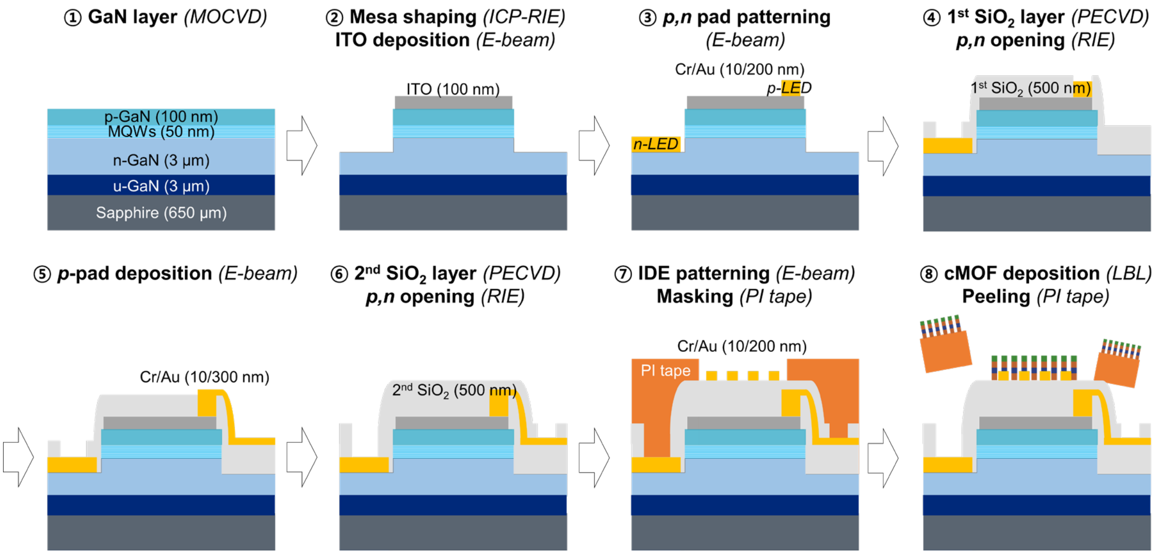

Supplementary Fig. 16 | Fabrication process of the photoactivated μLED gas sensor integrated with cMOF. The μLED was first fabricated using an MOCVD-grown GaN layer through a MEMS process. Then, a 1 μm thick SiO_2 layer was used to insulate the μLED, followed by the patterning of interdigitated electrodes (IDE). After covering the LED pad with polyimide (PI) tape, cMOF was synthesized on the IDE using a layer-by-layer method. The cMOF is photoactivated by the light emitted from the underlying μLED, enabling gas sensing.

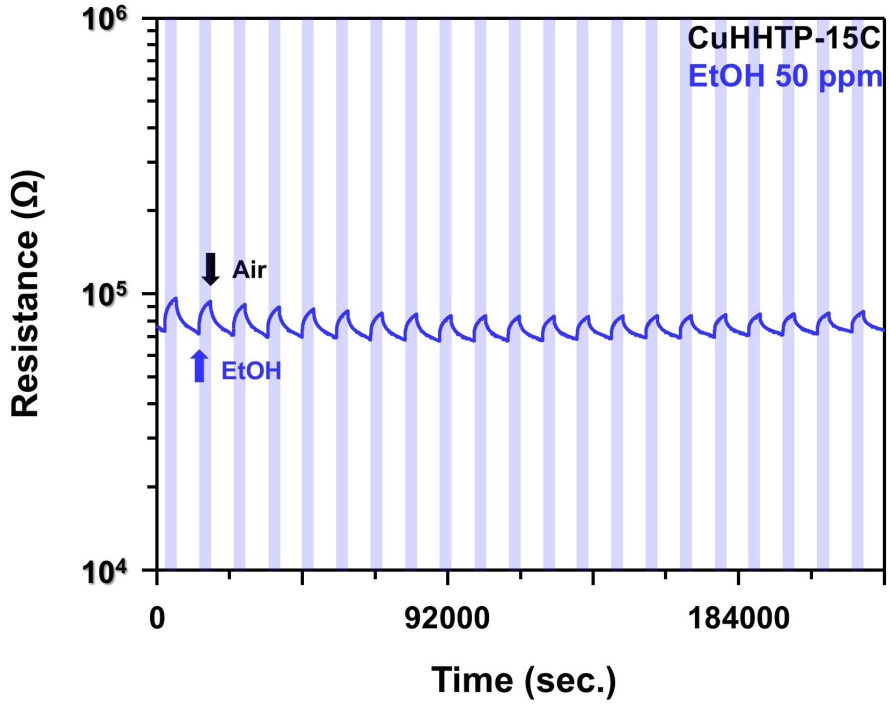

Supplementary Fig. 17 | Repeatability of the CuHHTP-15C sensor to 50 ppm of EtOH under external UV light source (response: 3600 s, recovery: 7200 s).

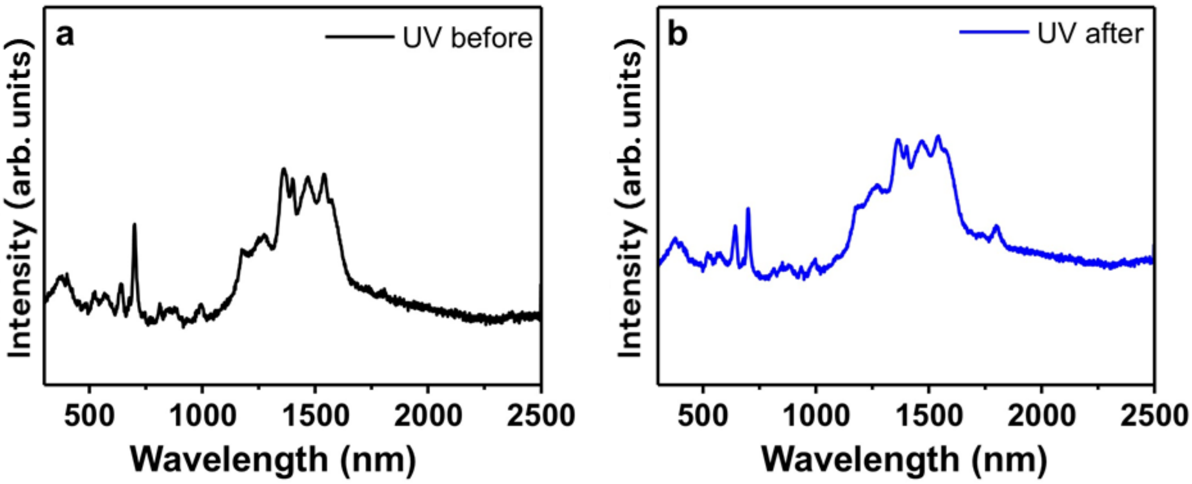

Supplementary Fig. 18 | a–b Raman spectroscopy (laser: 514 nm) results of CuHHTP-15C film of before (**a**) and after (**b**) 50 ppm EtOH repeatability test under UV light.

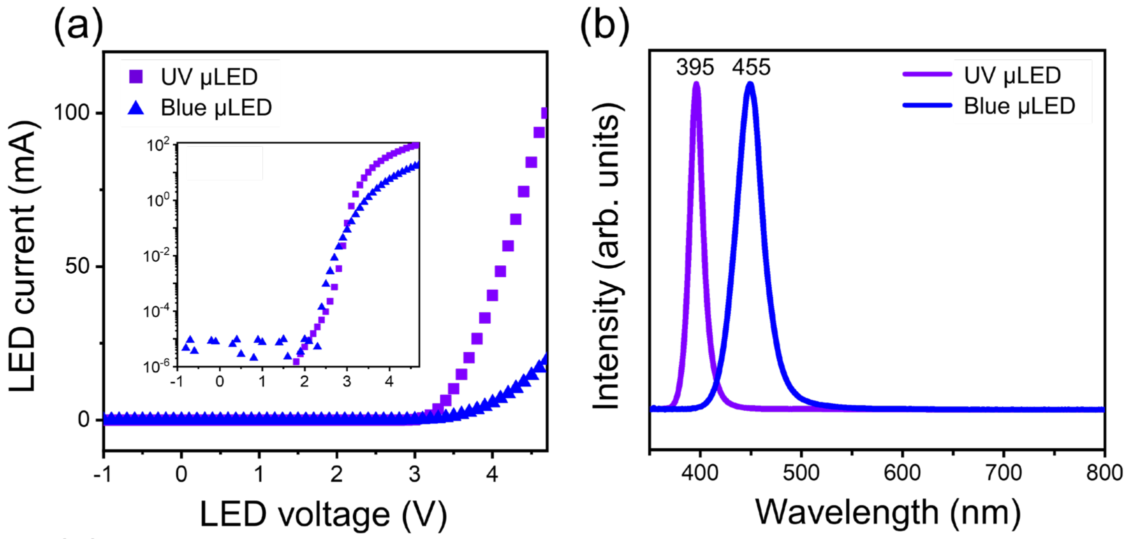

(c)

	Blue L1	Blue L2	Blue L3	UV L1
μLED voltage (V)	2.9	3.1	3.3	2.9
μLED current (μA)	21.8	84.0	262.0	22.8
μLED power (μW)	63.2	260.4	864.6	66.1

Supplementary Fig. 19 | a I-V curve of UV and blue μLEDs (inset: log scale). **b** Spectrum of UV and blue μLEDs. **c** Table summarizing the driving voltage, current, and power consumption of blue L1, L2, L3, and UV L1 at respective operating conditions.

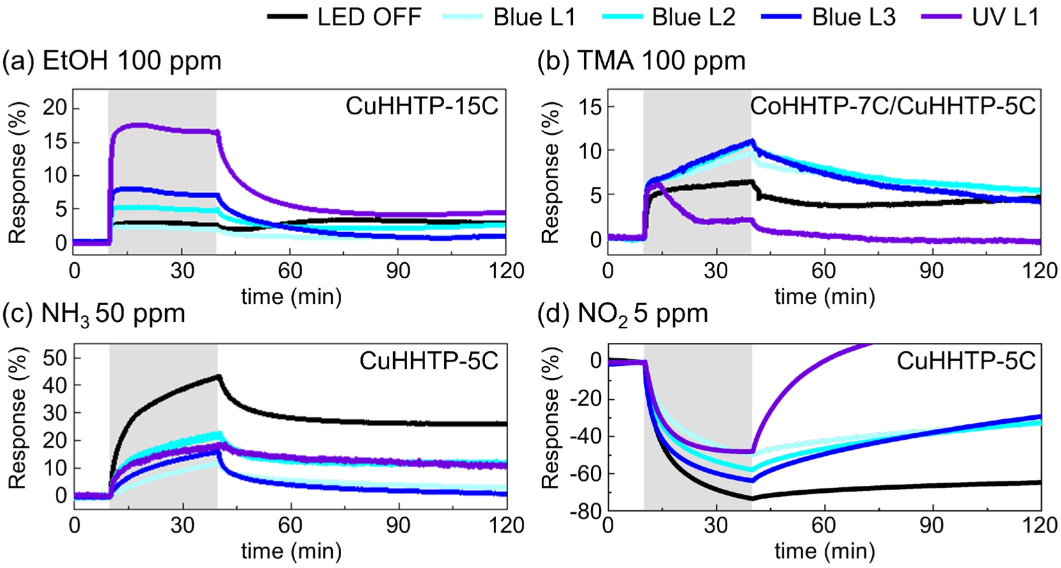

Supplementary Fig. 20 | a–d Gas sensing results for various µLEDs and light intensities with cMOF sensing layers optimized for each target gas. The values in parentheses indicate the best-performing µLED and light intensity. (**a**) EtOH + CuHHTP-15C (UV L1) (**b**) TMA + CoHHTP-7C/CuHHTP-5C (blue L2) (**c**) NH_3 + CuHHTP-5C (dark) (**d**) NO_2 + CuHHTP-5C (blue L2).

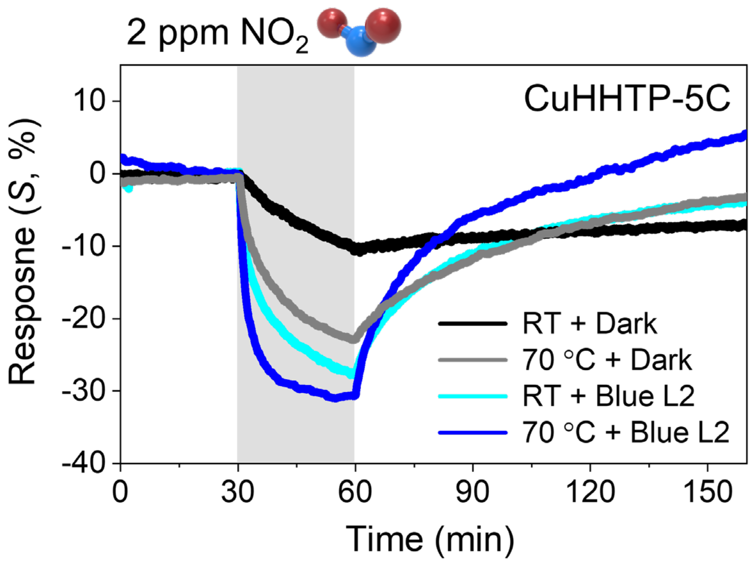

Supplementary Fig. 21 | Comparative study of the recovery behavior of the CuHHTP-5C sensor toward 2 ppm NO_2 under thermal and photoactivation conditions. The results demonstrate that photoactivation using the blue µLED (L2) is more effective in promoting recovery than thermal heating alone.

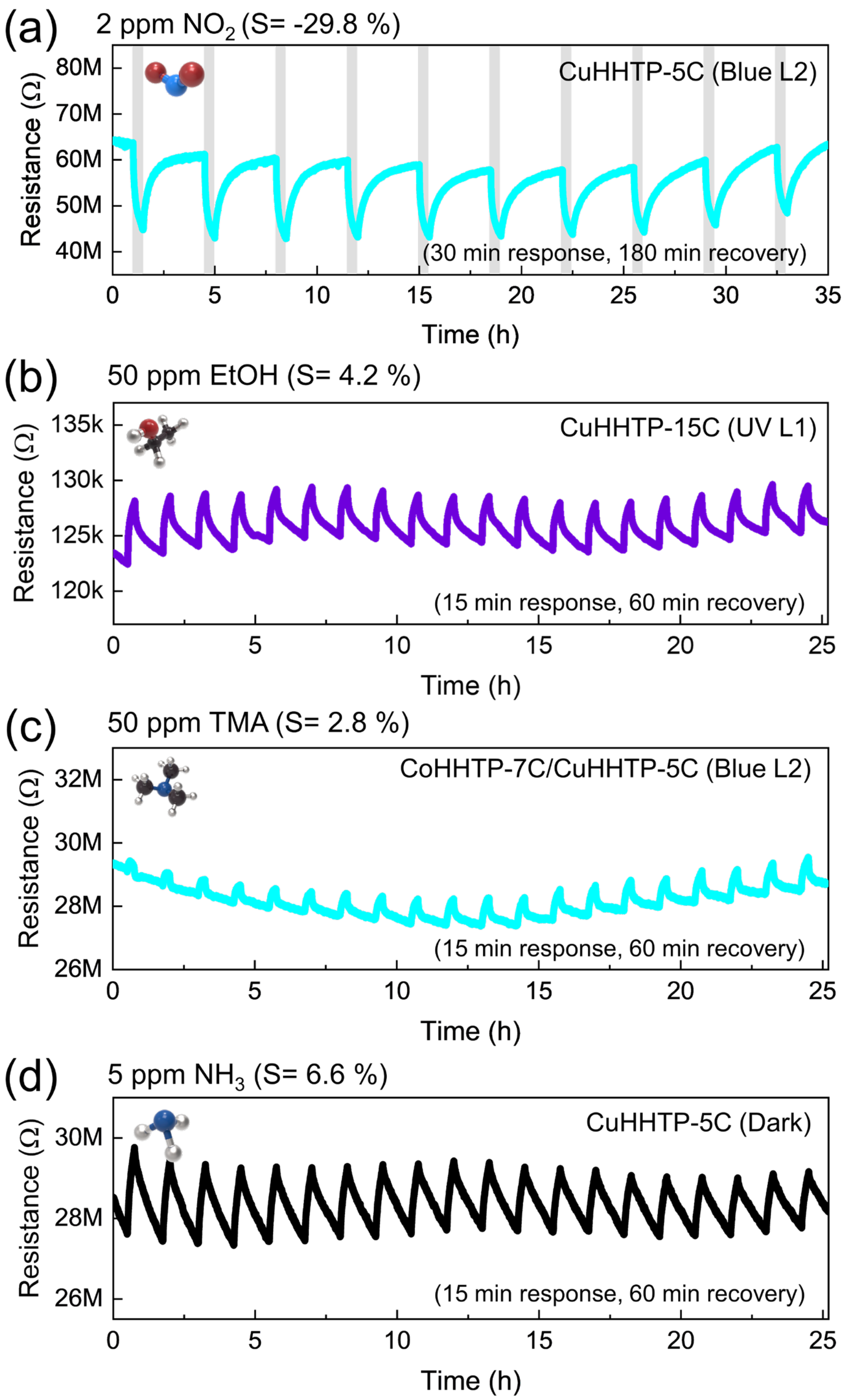

Supplementary Fig. 22 | a–d Repeatability test of optimized cMOF sensors under cyclic gas exposures. (**a**) CuHHTP-5C (Blue L2) sensor to 2 ppm NO_2 over 10 cycles. (**b**) CuHHTP-15C (UV L1) sensor to 50 ppm EtOH over 20 cycles. (**c**) CoHHTP-7C/CuHHTP-5C (Blue L2) sensor to 50 ppm TMA over 20 cycles. (**d**) CuHHTP-5C (Dark) sensor to 5 ppm NH_3 over 20 cycles.

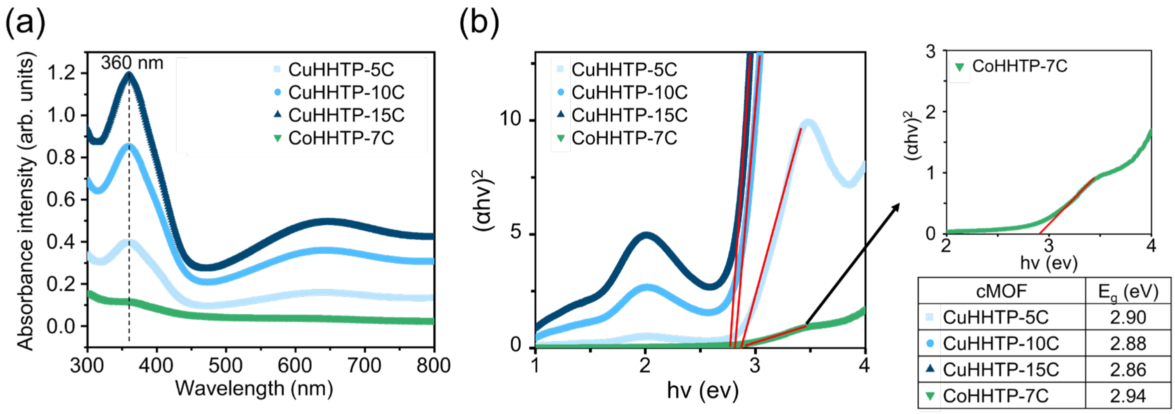

cMOF	E_g (eV)
CuHHTP-5C	2.90
CuHHTP-10C	2.88
CuHHTP-15C	2.86
CoHHTP-7C	2.94

Supplementary Fig. 23 | a–b (**a**) Absorbance spectra and (**b**) Tauc plot graphs of CuHHTP-5,10,15C and CoHHTP-7C measured using a UV-vis spectrophotometer. The UV-Vis spectrum exhibits two distinct peaks around 360 nm and 645 nm. The former corresponds to π–π* transition states with a relatively large energy gap (~2.88 eV), while the latter is associated with ligand-to-metal charge transfer (LMCT), which has a lower energy gap (~0.73 eV). Although both transitions can be excited under LED illumination (3.13 eV for UV LED and 2.72 eV for blue LED), the low-energy LMCT states are already significantly thermally activated, making them insufficient for generating additional charge carriers through photoexcitation. In contrast, photogenerated charges from the higher energy π–π* transitions predominantly contribute to the photogeneration of additional charges.

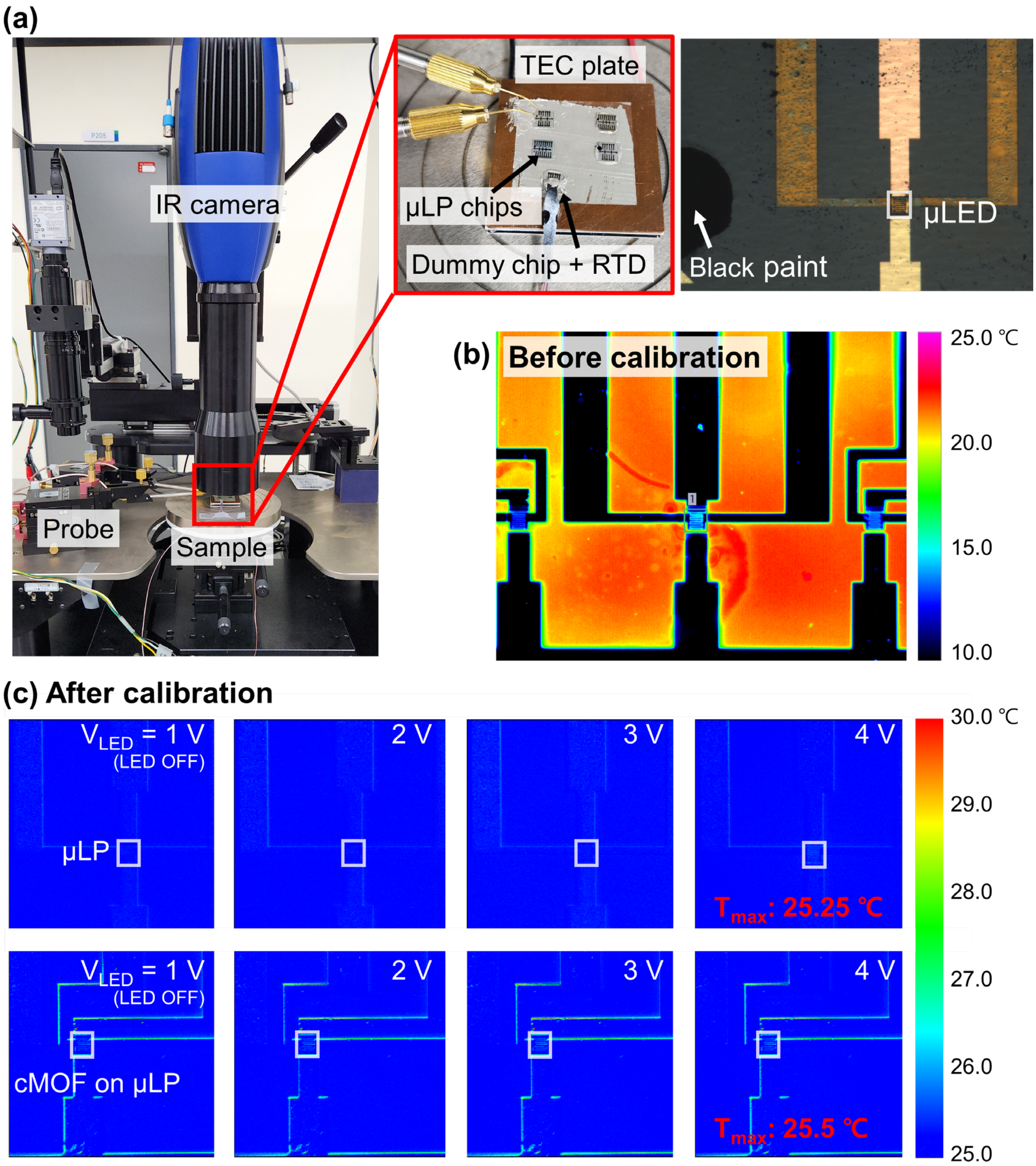

Supplementary Fig. 24 | a Infrared micro-thermography measurement setup for precise temperature measurement of the μLED and cMOF. **b** Temperature measurement image of the blue μLP before calibration. **c** Calibrated temperature measurement images of the blue μLP and the blue μLP integrated with CuHHTP-5C as forward bias is applied from 1V to 4V.

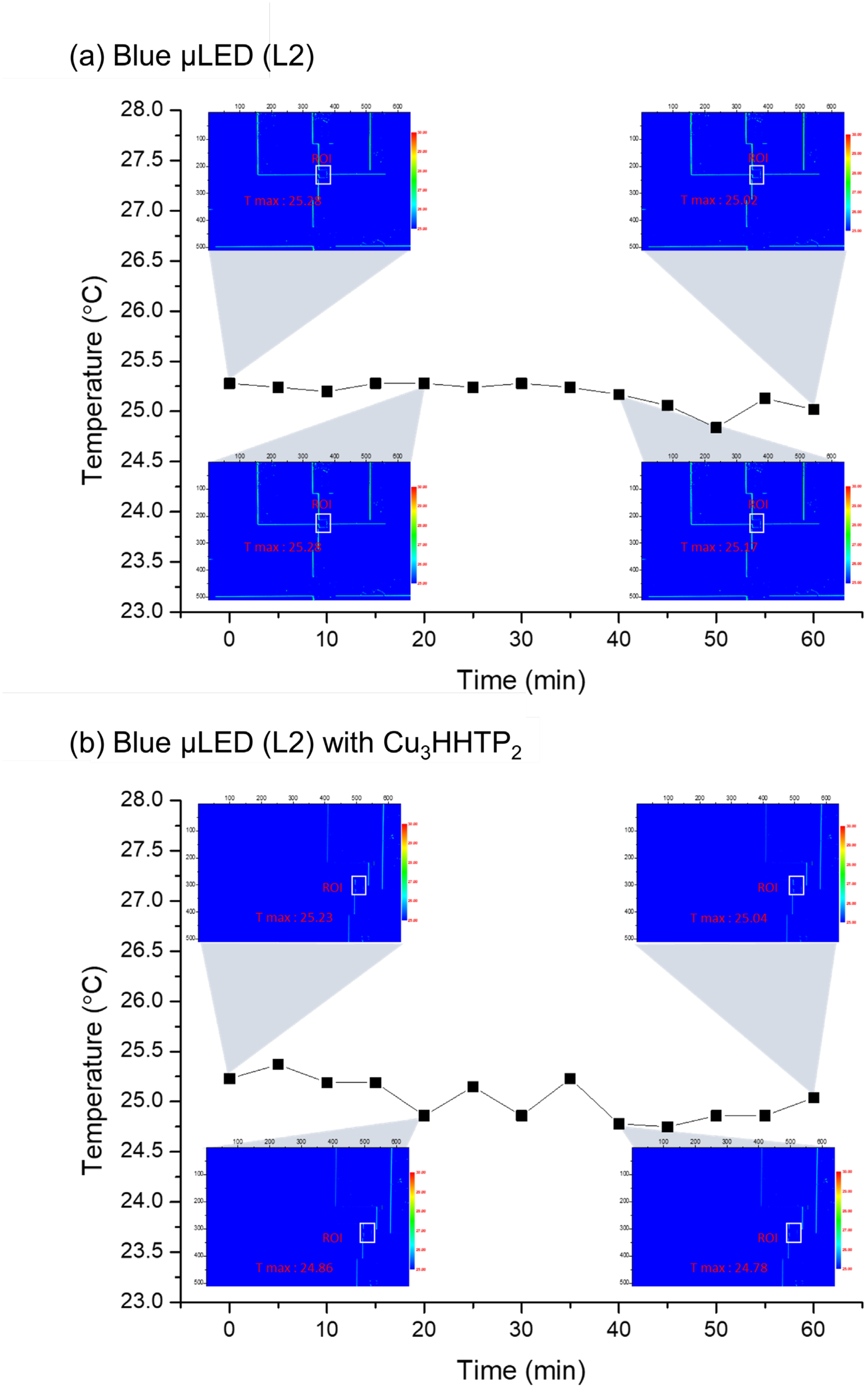

Supplementary Fig. 25 | a–b Calibrated temperature profiles under continuous L2 illumination. (**a**) Blue µLED-only sample. (**b**) Cu_3HHTP_2 synthesized on blue µLED sample.

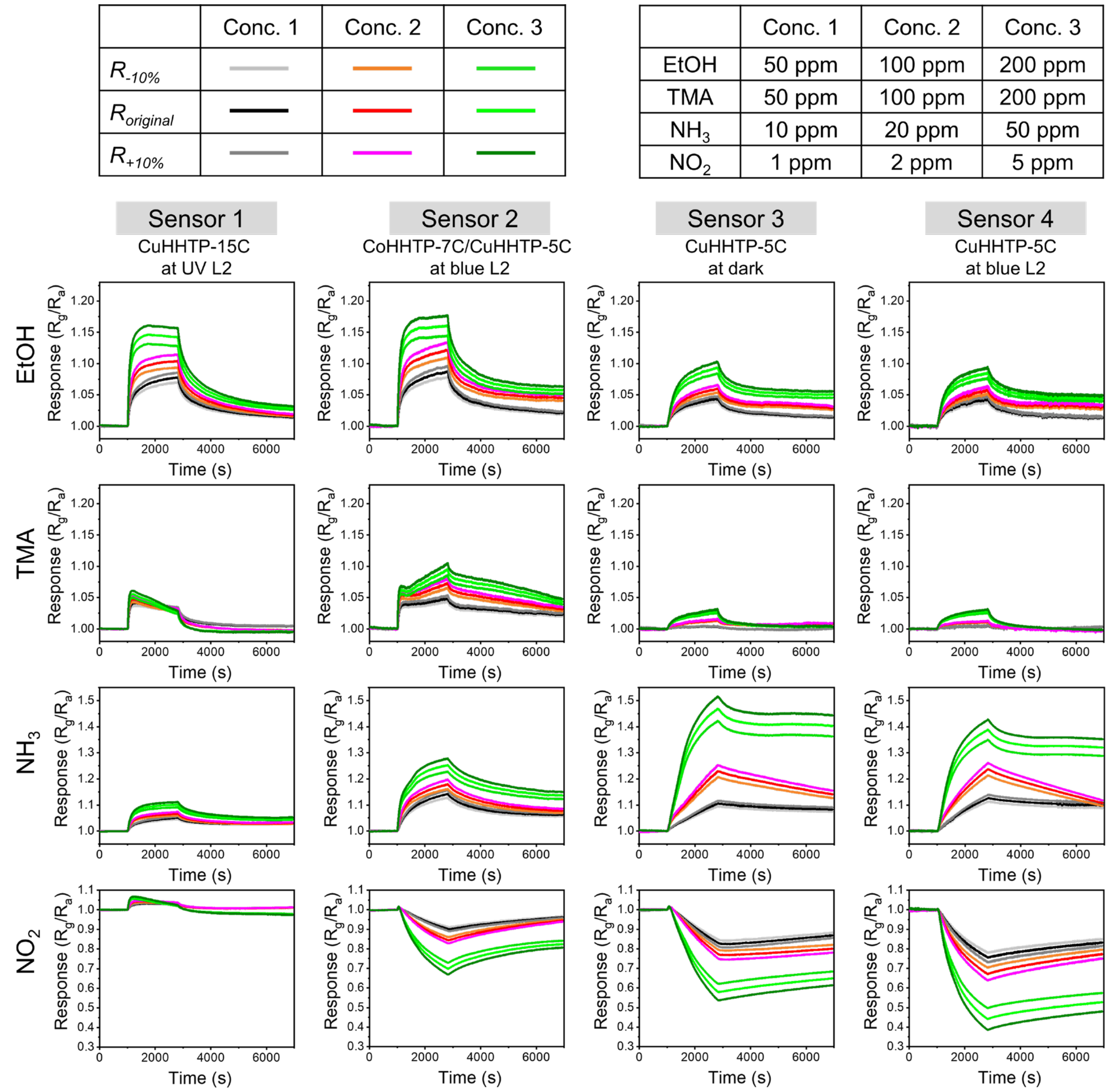

Supplementary Fig. 26 | Graph showing the gas response and recovery periods with +10% ($R_{+10\%}$) and -10% ($R_{-10\%}$) variations generated through data augmentation, compared with the original data (R_{original}).

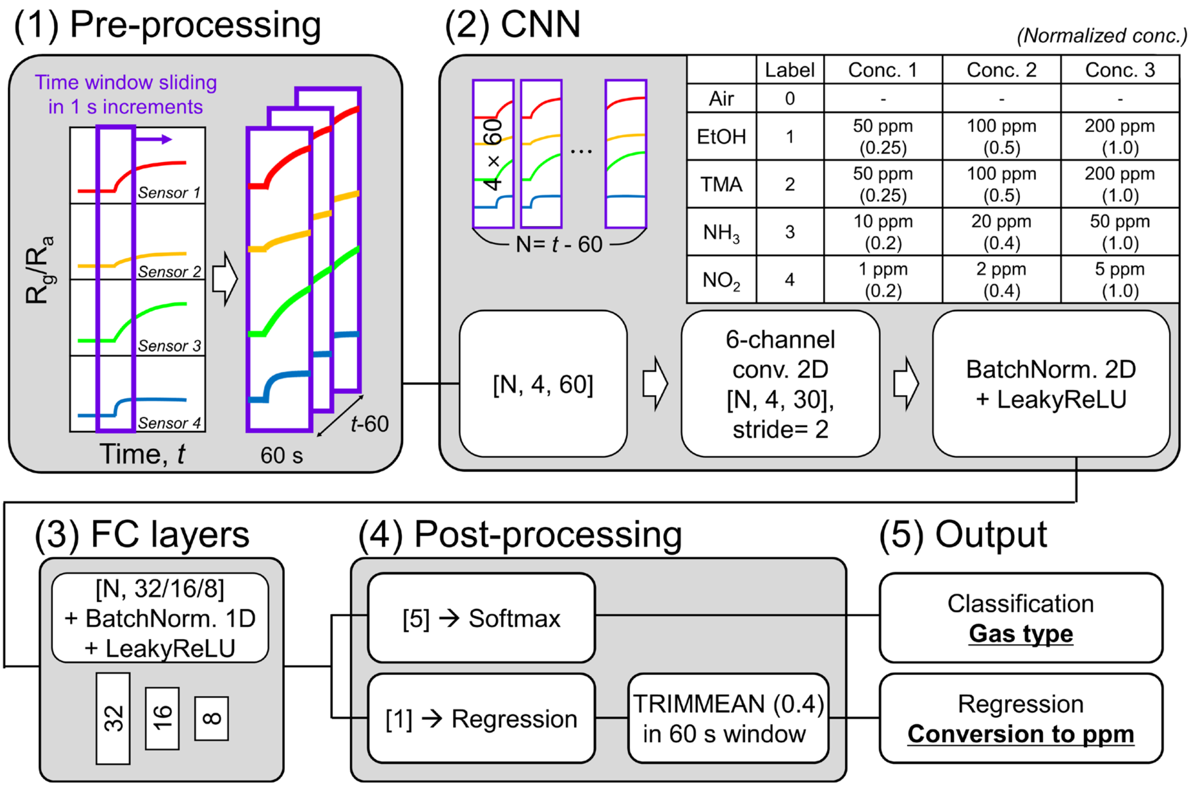

Supplementary Fig. 27 | The structure and training process of the e-nose system based on a chemiresistive cMOF sensor array.

Supplementary Table 1 | Response of CuHHTP-*x*C (*x* = 1, 3, 5, 7, 9, 11, 13, and 15) and their sample-to-sample deviations. The variation between samples decreases and stabilizes as the number of coating cycles increases. In the case of NO_2, the high deviation observed in coatings with more than 11 cycles is not due to instability of the sensing film but rather the low sensitivity, which increases the signal-to-noise ratio. All results represent the average values from different sensors (n= 2 to 4).

**Sensors**	**Response (%) [Sample-to-sample deviations]**			
	**EtOH 100 ppm**	**TMA 100 ppm**	**NH_3 5 ppm**	**NO_2 5 ppm**
CuHHTP-1C	12.32±2.05 [16.6%]	10.73±1.19 [11.1%]	35.96±4.86 [13.5%]	70.36±4.00 [5.7%]
CuHHTP-3C	17.74±2.98 [16.8%]	6.81±1.32 [19.5%]	20.25±0.87 [4.3%]	63.38±4.58 [7.2%]
CuHHTP-5C	23.11±1.24 [5.3%]	7.19±0.09 [1.2%]	12.32±0.49 [4.0%]	38.10±3.32 [8.7%]
CuHHTP-7C	24.43±0.23 [0.9%]	8.99±0.63 [7.1%]	9.53±0.28 [2.9%]	24.92±2.44 [9.8%]
CuHHTP-9C	23.39±0.42 [1.8%]	7.21±0.39 [5.3%]	9.25±0.14 [1.6%]	28.76±3.77 [13.1%]
CuHHTP-11C	21.23±0.61 [2.9%]	7.80±0.22 [2.8%]	6.51±0.25 [3.9%]	2.49±0.51 [20.7%]
CuHHTP-13C	21.72±0.59 [2.7%]	8.46±0.31 [3.7%]	5.94±0.39 [6.5%]	1.88±0.60 [32.2%]
CuHHTP-15C	22.20±0.21 [1.0%]	8.67±0.17 [1.9%]	4.91±0.20 [4.1%]	2.92±0.69 [23.48%]

Supplementary Table 2 | Prediction errors (mean absolute error; MAE) of the CNN model for gas types and concentrations.

Target gases							
EtOH		TMA		NH_3		NO_2	
50 ppm	11.1%	50 ppm	22.5%	10 ppm	10.0%	1 ppm	8.2%
100 ppm	8.1%	100 ppm	4.7%	20 ppm	6.1%	2 ppm	8.5%
200 ppm	2.7%	200 ppm	4.6%	50 ppm	3.2%	5 ppm	5.6%
